# Supplementary material for: Characterization of Naturally Occurring Bioactive Factor Mixtures for Bone Regeneration
Source: Int J Mol Sci. 2020 Feb 19;21(4):1412. doi: 10.3390/ijms21041412 (PMC7073126; doi:10.3390/ijms21041412)
Supplement: Supplementary file 1 [file ijms-21-01412-s001.pdf]

Supplementary Materials

Characterization of Naturally Occurring Bioactive Factor Mixtures for Bone Regeneration

Henriette Bretschneider [1,2], Mandy Quade [2], Anja Lode [2], Michael Gelinsky [2], Stefan Rammelt [1], Stefan Zwingenberger [1,2], Klaus-Dieter Schaser [1] and Corina Vater [1,2,*]

[1] University Center of Orthopaedics and Traumatology, University Hospital Carl Gustav Carus of Technische Universität Dresden, Fetscherstraße 74, 01307 Dresden, Germany; henriette.bretschneider@uniklinikum-dresden.de (H.B.), stefan.rammelt@uniklinikum-dresden.de (S.R.), stefan.zwingenberger@uniklinikum-dresden.de (S.Z.), klaus-dieter.schaser@uniklinikum-dresden.de (K.-D.S.)

[2] Centre for Translational Bone, Joint and Soft Tissue Research, University Hospital Carl Gustav Carus and Faculty of Medicine of Technische Universität Dresden, Fetscherstraße 74, 01307 Dresden, Germany; mandy.quade@tu-dresden.de (M.Q.), anja.lode@tu-dresden.de (A.L.), michael.gelinsky@tu-dresden.de (M.G.)

* Correspondence: corina.vater@uniklinikum-dresden.de

Table S1: Results of the proteome profiler array of 55 human angiogenesis-related proteins (Proteome Profiler Human Angiogenesis Array Kit, R&D, USA); - protein not detected; + lower, ++ middle, +++ upper third of measured proteins.

Table S2: Results of the proteome profiler array of 36 cyto- and chemokines (Proteome Profiler Human Cytokine Array Kit, R&D, USA); - chemokine not detected; + lower, ++ middle, +++ upper third of measured chemokines.

	PL	HCM	ATE
CCL2/MCP-1	-	+++	+++
CCL5/RANTES	++	-	-
Complement Component C5/C5a	+	++	++
CD40 Ligand/TNFSF5	+++	-	-
CXCL1/GROα	++	+	++
CXCL12/SDF-1	+	-	-
G-CSF	-	-	++
GM-CSF	-	+	+
ICAM-1/CD54	++	++	++
IL-1β/IL-1F2	-	-	++
IL-1ra/IL-1F3	-	-	++
IL-6	-	+++	+++
IL-8	-	+++	++
IL-16	+	-	+
IL-18/IL-1F4	+++	-	-
MIF	+	+++	++
Serpin E1/PAI-1	+	+++	++

	PL	HCM	ATE
Activin A	-	+	-
Angiogenin	+++	+++	+++
Angiopoietin-1	++	-	+
Angiopoietin-2	-	-	+++
Angiogenin	-	+++	-
Amphiregulin	-	+	+
Coagulation Factor III	-	+	++
CXCL16	-	++	++
DPPIV	++	+++	+++
EGF	+	-	-
Endoglin	++	++	-
Endostatin/Collagen XVIII	++	++	+++
Endothelin-1	-	++	-
FGF basic	+	-	+
FGF acidic	-	+	++
GDNF	-	+	+
GM.CSF	-	++	+++
HB-EGF	++	+	+
HGF	-	-	+
IGFBP-1	+++	+++	+++
IGFBP-2	++	++	++
IGFBP-3	++	++	+++
IL-1β	-	-	++
IL-8	-	+++	++
LAP (TGF-ß1)	++	+	-
Leptin	-	-	+++
MCP-1	-	+++	+++
MIP-1α	-	+	+
MMP-8	++	+	++
MMP-9	++	+	++
NRG1-β1	-	-	+
Pentaxin 3 (PTX3)	-	+++	+++
PD-ECGF	-	-	++
PDGF-AA	+++	-	+
PDGF-AB/PDGF-BB	++	-	-
Platelet Factor 4 (PF4)	-	+++	+++
PlGF	-	-	++
Prolactin	-	-	++
Serpin B5	-	+	+
Serpin E1	+	++	++
Serpin F1	+	++	++
TIMP-1	++	++	+++
TIMP-4	-	+	+++
Thrombospondin 1	-	+++	+++

Thrombospondin 2	-	++	+
µPA	+	+++	+++
Vasohibin	-	-	+
VEGF	++	++	+++
VEGF-C	-	-	+
